# Correction: Clinical Predictors of Survival for Patients with Stage IV Cancer Referred to Radiation Oncology

**DOI:** 10.1371/journal.pone.0130511

**Published:** 2015-06-19

**Authors:** Johnny Kao, Kenneth D. Gold, Gina Zarrili, Emily Copel, Andrew J. Silverman, Shanata S. Ramsaran, David Yens, Samuel Ryu

## Notice of Republication

This article was republished on May 26, 2015, to replace an incorrect Fig 1. Please download this article again to view the correct version. The originally published, uncorrected article and the republished, corrected article are provided here for reference.

## Supporting Information

S1 File Originally published, uncorrected article. (PDF)

S2 File Republished corrected article. (PDF)
