# Correction: Clinical Predictors of Survival for Patients with Stage IV Cancer Referred to Radiation Oncology

**DOI:** 10.1371/journal.pone.0132748

**Published:** 2015-07-13

**Authors:** Johnny Kao, Kenneth D. Gold, Gina Zarrili, Emily Copel, Andrew J. Silverman, Shanta S. Ramsaran, David Yens, Samuel Ryu

The sixth author’s name is spelled incorrectly. The correct name is: Shanta S. Ramsaran.
