# Supplementary material for: Correction: Clinical Predictors of Survival for Patients with Stage IV Cancer Referred to Radiation Oncology
Source: PLoS One. 2015 Jun 19;10(6):e0130511. doi: 10.1371/journal.pone.0130511 (PMC4474671; doi:10.1371/journal.pone.0130511)
Supplement: S1 File — (PDF) [file pone.0130511.s001.pdf]

PLOS ONE

RESEARCH ARTICLE

# Clinical Predictors of Survival for Patients with Stage IV Cancer Referred to Radiation Oncology

**Johnny Kao[1]*, Kenneth D. Gold[2], Gina Zarrili[3], Emily Copel[3], Andrew J. Silverman[1], Shanata S. Ramsaran[1], David Yens[4], Samuel Ryu[5]**

**1** Good Samaritan Hospital Medical Center, Department of Radiation Oncology, West Islip, New York, United States of America, **2** Good Samaritan Hospital Medical Center, Division of Hematology and Medical Oncology, West Islip, New York, United States of America, **3** Good Samaritan Hospital Medical Center, Division of Palliative Medicine, West Islip, New York, United States of America, **4** New York College of Osteopathic Medicine, Dept of Educational Development and Assessment, Old Westbury, New York, United States of America, **5** Stony Brook University Medicine, Department of Radiation Oncology, Stony Brook, New York, United States of America

* Johnny.kao@chsli.org

OPEN ACCESS

**Citation:** Kao J, Gold KD, Zarrili G, Copel E, Silverman AJ, Ramsaran SS, et al. (2015) Clinical Predictors of Survival for Patients with Stage IV Cancer Referred to Radiation Oncology. PLoS ONE 10(4): e0124329. doi:10.1371/journal.pone.0124329

**Academic Editor:** Qing-Yi Wei, Duke Cancer Institute, UNITED STATES

**Received:** January 20, 2015

**Accepted:** March 2, 2015

**Published:** April 20, 2015

**Copyright:** © 2015 Kao et al. This is an open access article distributed under the terms of the Creative Commons Attribution License, which permits unrestricted use, distribution, and reproduction in any medium, provided the original author and source are credited.

**Data Availability Statement:** To ensure compliance with the existing IRB approval letter and HIPAA compliance for patients, the full deidentified dataset cannot be made available without prior written approval from the Good Samaritan Hospital Institutional Review Board. Requests for deidentified data can be made to Dr. William Engellener, Chairman of the Good Samaritan Hospital Medical Center Institutional Review Board.

**Funding:** The author(s) received no specific funding for this work.

## Abstract

### Background

There is an urgent need for a robust, clinically useful predictive model for survival in a heterogeneous group of patients with metastatic cancer referred to radiation oncology.

### Methods

From May 2012 to August 2013, 143 consecutive patients with stage IV cancer were prospectively evaluated by a single radiation oncologist. We retrospectively analyzed the effect of 29 patient, laboratory and tumor-related prognostic factors on overall survival using univariate analysis. Variables that were statistically significant on univariate analysis were entered into a multivariable Cox regression to identify independent predictors of overall survival.

### Results

The median overall survival was 5.5 months. Four prognostic factors significantly predicted survival on multivariable analysis including ECOG performance status (0–1 vs. 2 vs. 3–4), number of active tumors (1 to 5 vs. $\geq$6), albumin levels ($\geq$3.4 vs. 2.4 to 3.3 vs. <2.4 and primary tumor site (Breast, Kidney or Prostate vs. Other). Risk group stratification was performed by assigning points for adverse prognostic factors resulting in very low, low, intermediate and high risk groups. The median survival was >31.4 months for very low risk patients compared to 14.5 months for low risk, 4.1 months for intermediate risk and 1.2 months for high risk (p<0.001).

**Competing Interests:** The authors have declared that no competing interests exist

## Conclusions

These data suggest that a model that considers performance status, extent of disease, primary tumor site and serum albumin represents a simple model to accurately predict survival for patients with stage IV cancer who are potential candidates for radiation therapy.

## Introduction

Approximately half of cancer patients referred for radiotherapy evaluation have stage IV cancer [1]. An accurate estimation of life expectancy of patients with metastatic cancer remains a difficult challenge for clinicians [2]. When using intuition and experience alone, clinicians systematically overestimate survival in patients with incurable cancer [3,4]. A more accurate estimate of survival can reduce the administration of unnecessarily protracted courses of palliative radiotherapy [5,6]. Conversely, it is important for clinicians to identify a subset of patients with metastatic cancer that can benefit from improved local control and disease-free survival with aggressive local and systemic therapy [7,8]. Therefore, there is a clear need for robust models of predicted survival in stage IV cancer.

Patients with metastatic cancer are heterogeneous and with the exception of performance status, there has not been uniform agreement on predictors of survival [9]. Performance status alone only accounts for less than half of the variability in survival observed in terminally ill cancer patients [10]. A recent review by the European Association for Palliative Care of published studies attempted to identify favorable and unfavorable subgroups and showed that prognostic factors evaluated were highly variable [11]. In general, performance status and clinical signs and symptoms of organ failure, including dyspnea, dysphagia, weight loss, anorexia and altered mental status, were the strongest predictors of survival [11]. Although not yet widely used in radiation oncology, the Palliative Performance Scale Score augments performance status with measures of extent of disease, self-care, oral intake and level of consciousness [12,13]. In recent studies investigating patients who were still candidates for anticancer therapy, there was additional value in incorporating tumor type, extent of metastatic disease and select laboratory values to further refine the prognostic model [14,15].

With the goal of improving estimates of survival among patients with stage IV cancer referred to radiation oncology, we performed a comprehensive analysis that systematically evaluates possible clinical, imaging, laboratory and pathologic predictors of survival.

## Materials and Methods

### Inclusion Criteria

This study included consecutive patients older than 18 years with metastatic stage IV solid tumor who were referred to a single physician in a large community hospital-based radiation oncology department. This minimal risk study was approved by the Good Samaritan Hospital Institutional Review Board with waiver of informed consent.

### Data Collection

The history, physical examination, radiologic studies, pathology and laboratory tests were documented by review of electronic medical record (EPIC). Confirmation of survival was performed by review of most recent office visit or confirmed activity in the hospital electronic medical record. Confirmation of survival status and date of death was performed using review

of the Social Security Death Index. Patients who were lost to follow-up were censored at the last clinic visit.

The following patient-related factors were collected: age, gender, race, ECOG performance score, weight loss, marital status, Charlson comorbidity score and symptoms (dyspnea, pain, altered mental status, symptoms of anorexia/cachexia syndrome) [1,9,16,17]. Altered mental status is defined as change in brain function from baseline including confusion, drowsiness, delirium, dementia or coma. Laboratory examination included leukocyte count, percentage of lymphocytes, serum albumin and total bilirubin [18,19,20]. Lactate dehydrogenase and C reactive protein were not routinely performed at our institution. The following tumor related factors were analyzed: primary tumor site, histology, metastasis site(s), number of active tumors, number of involved organs, disease status (newly diagnosed with stage IV vs. prior diagnosis of stage IV cancer) and disease-free interval $>$12 months [1,9]. Number of active tumors was quantified by identifying tumors measuring $>$ 1 cm in short axis on CT or MRI or increased radiotracer activity on PET or bone scan. Oligometastases were defined as 1 to 5 active tumors on whole body imaging [21]. Breast, prostate and kidney cancers have previously been associated with a more favorable prognosis in patients with metastatic disease receiving radiotherapy [15,22]. There was no missing data with the exception of marital status and laboratory values in $\leq$20% of the patient population.

## Statistical Methodology

Statistical analysis was performed with Stata 8.0. The primary outcome was overall survival, defined as time from initial radiation oncology consultation to date of death. Survival data was analyzed using the Kaplan-Meier method and summarized by median and 6-month survival. The log-rank method was used to compare the effect of patient and tumor-related variables on overall survival. Continuous variables were categorized into two or three classes using cut points suggested by literature review.

To adjust for the effects of multiple comparisons, variables that were significant at a p value of 0.02 after univariate analysis were entered into a Cox multivariable analysis. The proportional hazards assumption were graphically checked by examining log[-log(probability)] plot over time. To determine the prognostic value of each covariate in the final model, a linear regression was performed on 6-month survival. The proportion of variability in observed survival that was explained by the predicative model was measured by the multiple correlation coefficient $R^2$.

# Results

## Patient-Specific Prognostic Factors

Between May 2012 to September 2013, 143 patients with distant metastases were referred for radiation oncology evaluation with 57% inpatient consultations and 43% outpatient consultations. The median survival was 5.5 months. The median follow-up for surviving patients was 19.1 months (range 0.5 to 32.1 months). The mean age was 67 years +/- 13 years (range 33 to 97). Women accounted for 59% of the patient population and the majority of patients were white (80%). With respect to performance status, 29% were ECOG 0–1, 32% were ECOG 2 and 40% were ECOG 3–4. The incidence of moderate to severe dyspnea was 15% and the incidence of moderate to severe pain was 48%. Altered mental status was observed in 14%, extensive comorbidity was noted in 8% and significant weight loss, anorexia or dysphagia was noted in 34% (Table 1).

We performed univariate analysis on the effect of patient factors on survival. Gender, race, dyspnea, pain and serum bilirubin did not predict survival. Nine factors strongly predicted short survival including age $\geq$80, ECOG 3–4, not married status, altered mental status,

Table 1. Univariate analysis of patient characteristics on survival in patients with advanced cancer.

| Variable | Number (%) | P | Median Survival (months) | 6 month survival |
|---|---|---|---|---|
| **Overall population** | | | **5.5** | **48%** |
| **Age** | | <0.001 | | |
| <60 | 37 (26%) | | 10.2 | 66% |
| 60 to 79 | 74 (52%) | | 5.7 | 49% |
| ≥80 | 32 (22%) | | 1.5 | 26% |
| **Gender** | | 0.93 | | |
| Male | 58 (41%) | | 4.2 | 46% |
| Female | 85 (59%) | | 5.7 | 50% |
| **Race** | | | | |
| White | 114 (80%) | 0.07 | 6.5 | 53% |
| Non-white | 29 (20%) | | 4.2 | 28% |
| **ECOG performance** | | | | |
| 0–1 | 41 (29%) | <0.001 | 21.6 | 90% |
| 2 | 44 (31%) | | 5.7 | 48% |
| 3 | 45 (31%) | | 1.7 | 23% |
| 4 | 13 (9%) | | 0.5 | 8% |
| **Marital status** | | 0.002 | | |
| Married | 64 (45%) | | 9.7 | 65% |
| Not married | 58 (41%) | | 4.1 | 37% |
| Unknown | 21(15%) | | | |
| **Dyspnea** | | 0.33 | | |
| Borg 0 to 2 | 122 (85%) | | 5.2 | 48% |
| Borg ≥3 | 21 (15%) | | 5.5 | 51% |
| **Pain** | | 0.82 | | |
| Pain scale 0 to 4 | 74 (52%) | | 5.2 | 46% |
| Pain ≥5 | 69 (48%) | | 5.2 | 51% |
| **Altered mental status** | | <0.001 | | |
| No | 123 (86%) | | 6.5 | 55% |
| Yes | 20 (14%) | | 1.4 | 10% |
| **Charlson comorbidity score** | | 0.001 | | |
| 0 to 3 | 132 (92%) | | 6.1 | 52% |
| ≥4 | 11 (8%) | | 0.7 | 9% |
| **Weight loss ≥ 10% or Anorexia or Dysphagia** | | <0.001 | | |
| No | 94 (66%) | | 8.2 | 61% |
| Yes | 49 (34%) | | 1.7 | 27% |
| **Elevated WBC** | | 0.003 | | |
| ≤11 | 88 (62%) | | 6.2 | 52% |
| >11 | 39 (27%) | | 2.7 | 28% |
| Unknown | 16 (11%) | | | |
| **Lymphopenia** | | <0.001 | | |
| ≥12% | 69 (48%) | | 8.2 | 59% |
| <12% | 53 (37%) | | 1.8 | 25% |
| Unknown | 21 (15%) | | | |
| **Albumin** | | <0.001 | | |
| ≥3.4 | 54 (47%) | | 9.7 | 61% |
| 2.4 to 3.3 | 52 (45%) | | 2.7 | 26% |
| <2.4 | 10 (9%) | | 1.5 | 10% |

*(Continued)*

**Table 1.** (*Continued*)

| Variable | Number (%) | P | Median Survival (months) | 6 month survival |
|---|---|---|---|---|
| **Overall population** | | | **5.5** | **48%** |
| Unknown | 27 (19%) | | | |
| **Bilirubin** | | 0.12 | | |
| Normal | 104 (73%) | | 4.6 | 41% |
| Elevated | 11 (8%) | | 1.7 | 36% |
| Unknown | 28 (20%) | | | |

doi:10.1371/journal.pone.0124329.t001

Charlson comorbidity score ≥4, weight loss, anorexia or dysphagia, white blood cell count >11, percent lymphocytes <12%, serum albumin 2.4 to 3.3 and serum albumin <2.4.

## Tumor-Specific Prognostic Factors

The most common primary tumor sites were lung (45%), breast (13%), colorectal (10%), prostate (6%), endometrial (5%), kidney (4%) and gastroesophageal (4%). The majority of tumors were adenocarcinoma (57%) followed by small cell carcinoma (11%) and squamous cell carcinoma (8%). One quarter of patients (25%) had 1 to 5 active tumors and 29% had only 1 involved organ. Cerebral metastases were present in 40% of patients, 50% had bone metastases, 20% had liver metastases, 47% had lung or pleural metastases, 39% had distant lymph nodes, 10% had adrenal metastases, 8% of patients had spinal cord compression or involvement, 5% had skin or muscle metastases and 9% had abdominal carcinomitosis or serosa/omental metastases.

The results of univariate analysis of tumor factors are listed in Table 2. Newly diagnosed cancer and time from initial diagnosis of cancer did not predict for survival while tumor size was a relatively weak predictor of survival (p = 0.02). Breast, prostate and kidney primary tumor, adenocarcinoma, 1 to 5 active tumors, single involved organ and absence of liver or spinal cord involvement were associated with longer survival. Bone only metastases weakly predicted survival (p = 0.03).

## Multivariable Analysis

Among 16 variables that were significant on univariate analysis, 4 remained significant on Cox multivariable analysis (Table 3). The strongest predictors of longer survival were ECOG performance status 0 to 1 (HR 1.95), fewer than 6 active tumors (HR 2.70), favorable primary tumor site (HR 3.33) and normal serum albumin (HR 2.09). There was a trend towards improved survival with normal mental status and adenocarcinoma histology, although these covariates did not reach statistical significance.

## Development of a Predictive Model

To obtain a composite score, we incorporated variables that were statistically significant on Cox multivariable analysis into a logistical regression to predict 6-month survival (Table 4). Evaluation of the regression coefficient for each covariate informed weighting of the final model. The relative magnitude of regression coefficients suggested that the two covariates with 2 subgroups (number of active tumors and favorable primary tumor site) should be weighed equally assigning 1 point for an unfavorable risk factor. For the two covariates with 3 subgroups (ECOG performance status and serum albumin), ECOG performance status had twice

**Table 2. Univariate analysis of tumor characteristics on overall survival of patients with advanced cancer.**

| Variable | Number (%) | P | Median Survival (months) | |
|---|---|---|---|---|
| **Primary tumor site** | | | | |
| Lung | 65 (45%) | | 4.6 | 43% |
| Breast | 19 (13%) | 0.003 (Breast vs. non-breast) | Not reached | 77% |
| Colorectal | 14 (10%) | | 6.3 | 70% |
| Prostate | 9 (6%) | | 8.0 | 78% |
| Uterus | 7 (5%) | | 3.8 | 43% |
| Kidney | 6 (4%) | | 11.6 | 83% |
| Esophagus/Gastric | 5 (4%) | | 1.5 | 0% |
| Pancreas | 4 (3%) | | 1.5 | 25% |
| Unknown Primary | 4 (3%) | | 0.7 | 25% |
| Other (Melanoma, Vulva, Cervix, Ovary, Sarcoma, Bladder, Salivary Gland, Thyroid) | 10 (7%) | | 0.7 | 0% |
| **Favorable Primary Site** | | <0.001 | | |
| Breast, Prostate or Kidney | 34 (24%) | | 16.5 | 78% |
| Others | 109 (76%) | | 4.1 | 39% |
| **Histology** | | | | |
| Adenocarcinoma | 81 (57%) | 0.001 (Adenocarcinoma vs. Other) | 8.0 | 60% |
| Small Cell Carcinoma | 16 (11%) | | 2.9 | 38% |
| Squamous Cell Carcinoma | 12 (8%) | | 2.7 | 33% |
| Poorly Differentiated or Carcinoma NOS | 11 (8%) | | 1.7 | 27% |
| Other (Renal Cell Carcinoma, Papillary Serous Carcinoma, Melanoma, Sarcoma, Urothelial Carcinoma, Adenoid Cystic Carcinoma, Mucinous Carcinoma, Carcinoid) | 16 (11%) | | 2.3 | 47% |
| No Biopsy | 7 (2%) | | 0.6 | 0% |
| **Largest Tumor Size** | | 0.02 | | |
| ≤5 cm | 73 (51%) | | 6.3 | 55% |
| 5.1 to 10 cm | 59 (41%) | | 4.1 | 42% |
| ≥10.1 cm | 11 (8%) | | 2.1 | 36% |
| **Number of Active Tumors** | | <0.001 | | |
| 1 | 10 (7%) | | 21.6 | 69% |
| 2 to 5 | 26 (18%) | | 12.7 | 79% |
| ≥6 | 107 (75%) | | 3.9 | 38% |
| **Number of Organs Involved** | | 0.001 | | |
| 1 | 41 (29%) | | 13.2 | 70% |
| ≥2 | 102 (71%) | | 4.2 | 40% |
| **Newly Diagnosed with Cancer** | | 0.99 | | |
| Yes | 87 (61%) | | 4.6 | 48% |
| No | 56 (39%) | | 5.7 | 49% |
| **Time from Initial Diagnosis of Cancer >12 Months** | | 0.91 | | |
| Yes | 43 (30%) | | 5.7 | 50% |
| No | 100 (70%) | | 4.6 | 48% |
| **Brain Metastases** | | 0.13 | | |
| Yes | 57 (40%) | | 4.2 | 40% |
| No | 86 (60%) | | 6.3 | 54% |
| **Liver Metastases** | | 0.004 | | |
| Yes | 29 (20%) | | 3.7 | 41% |

*(Continued)*

**Table 2.** (*Continued*)

| Variable | Number (%) | P | Median Survival (months) | |
|---|---|---|---|---|
| No | 114 (80%) | | 6.0 | 50% |
| **Spinal Cord Compression or Leptomeningeal Spread** | | <0.001 | | |
| Yes | 11 (8%) | | 0.9 | 9% |
| No | 132 (92%) | | 6.2 | 52% |
| **Bone only metastases** | | 0.03 | | |
| Yes | 12 (9%) | | Not reached | 75% |
| No | 130 (91%) | | 5.1 | 46% |

doi:10.1371/journal.pone.0124329.t002

the weight of albumin. Therefore, 1 point was assigned for ECOG 2, 2 points were assigned for ECOG 3 to 4, 0.5 points for serum albumin 2.4 to 3.3 and 1 point for serum albumin <2.4. Favorable covariates were assigned 0 points.

## Performance of the predictive model

Composite scores ranged from 0 to 5. Composite scores were used to classify patients into 4 groups with clearly distinct median survivals (Table 5). There was a very low risk cohort with a composite score of 0 to 1 had a median survival of >31.4 months (95% confidence interval 15.5 months to not reached). The low risk cohort with a composite score of 1.5 to 2 had a median survival of 14.5 months (95% confidence interval 8.0 to 21.4 months). The intermediate risk group had a composite score of 2.5 to 3.5 had a median survival of 4.1 months (95% confidence interval 2.6 to 5.7 months). The high risk group had a composite score of 4 to 5 and had a median survival of 1.2 months (95% confidence interval 0.7 to 1.5 months). Risk group strongly predicted overall survival with a p value of <0.001 (Fig 1).

Comparison of actual survival with predicted survival gave an $R^2$ value of 0.50 compared to 0.34 for ECOG performance status alone, 0.15 serum albumin, 0.11 for number of active tumors and primary tumor site.

# Discussion

After a comprehensive review of contemporary patients evaluated in a hospital-based radiation oncology center, we identified numerous predictors of survival on univariate and multivariate analysis. Importantly, we identified fairly common key subgroups of patients with a median survival of less than 2 months including age≥80, ECOG 3–4, lymphopenia and symptoms of anorexia/cachexia syndrome. Further, we identified less common subgroups of patients with median survival of less than 2 months including altered mental status, Charlson comorbidity score ≥4, serum albumin <2.4, elevated bilirubin, esophagus/gastric/pancreatic or unknown primary tumor, poorly differentiated carcinoma and spinal cord compression. For instance, a patient with ECOG 3–4 and ≥6 active tumors has a grave prognosis with a median survival of

**Table 3. Cox multivariable analysis of predictors of overall survival.**

| Variable | Hazard Ratio | 95% Confidence Interval | P value |
|---|---|---|---|
| ECOG Performance Status (0–1 vs. 2 vs. 3–4) | 1.95 | 1.25 to 3.03 | 0.003 |
| Number of Active Tumors (1 to 5 vs. ≥6) | 2.70 | 1.40 to 5.19 | 0.003 |
| Serum albumin (≥3.4 vs. 2.4 to 3.3 vs. <2.4) | 2.09 | 1.25 to 3.48 | 0.005 |
| Tumor Site (Breast, Kidney or Prostate vs. Other) | 3.33 | 1.27 to 8.76 | 0.015 |

doi:10.1371/journal.pone.0124329.t003

**Table 4. Linear regression of predictors of 6-month survival.**

| Variable | Regression Coefficient | 95% Confidence Interval | P value |
|---|---|---|---|
| ECOG Performance Status (0–1 vs. 2 vs. 3–4) | 0.26 | 0.17 to 0.36 | <0.001 |
| Number of Active Tumors (1 to 5 vs. ≥6) | 0.29 | 0.13 to 0.45 | 0.001 |
| Serum albumin (≥3.4 vs. 2.4 to 3.3 vs. <2.4) | 0.14 | 0.02 to 0.25 | 0.017 |
| Tumor Site (Breast, Kidney or Prostate vs. Other) | 0.31 | 0.12 to 0.50 | 0.002 |

doi:10.1371/journal.pone.0124329.t004

1.2 months and survival beyond 6 months is highly unlikely. Unfavorable risk patients may be best classified as far advanced metastatic disease.

Conversely, these data confirm that oligometastases are not uncommon, representing 25% of stage IV patients referred to radiation oncology [23]. Patients with ECOG 0–1, breast or kidney cancer, 1 to 5 active tumors and single organ involvement were associated with median survival greater than 12 months. Along with recently published biological and genetic studies, these data support the notion that patients with oligometastases are a distinct subset of stage IV cancers that can have prolonged disease-free survival [24]. Recent research suggests that stereotactic body radiotherapy with or without concurrent systemic therapy holds promise for further improving progression-free survival with an acceptable toxicity profile for selected patients with oligometastases [21,25,26].

In our study, we identified 4 robust predictors of survival on multivariable analysis. Consistent with prior studies, ECOG performance status was a strong predictor of survival but accounts for only a fraction of observed survival. Number of active tumors was also a strong predictor of survival that is also included in the Palliative Performance Scale Scores [12]. Anorexia/cachexia syndrome is a strong predictor of terminal cancer. In this study, low serum albumin strongly predicted for short survival. Somewhat surprising was that brain metastases did not strongly impact survival. In our series, 27% of patients with brain metastases survived over 12 months. There has been increasing interest in reducing the late toxicity of whole brain radiotherapy in long-term survivors of brain metastases [27]. However, since only 41% of brain metastases survive 6 months, careful selection using criteria such as ECOG 0–2, limited extracranial disease, no weight loss and no altered mental status for surgery and/or stereotactic radiosurgery.

The group from Toronto-Sunnybrook Regional Cancer Centre has published landmark studies on prognostic factors for a large population of patients with stage IV cancer referred to a palliative radiotherapy service [15]. With the goal of simplicity, they demonstrated that breast cancer, bone only metastases and KPS≥70 was associated with improved survival. However, the authors acknowledged that their statistically significant model explained less than 30% of the observed variability. A second study from University of Minnesota identified performance status as the strongest predictor of survival with some contribution from primary site and solitary metastasis for longer survivors ≥8 month [28]. In our analysis, patients with either primary breast, prostate or kidney primary tumors had better survival than other primary tumors.

**Table 5. Median and 6-month survival stratified by risk group.**

| Risk Score | Number (%) | Median survival | 6 month survival (95% confidence interval) |
|---|---|---|---|
| Very Low Risk (0 to 1) | 22 (15%) | Not reached | 100% (n/a) |
| Low Risk (1.5 to 2) | 30 (21%) | 14.5 months | 89% (69 to 96%) |
| Intermediate Risk (2.5 to 3.5) | 49 (34%) | 4.1 months | 37% (23 to 50%) |
| High risk (4 to 5) | 42 (29%) | 1.2 months | 7% (2 to 17%) |

doi:10.1371/journal.pone.0124329.t005

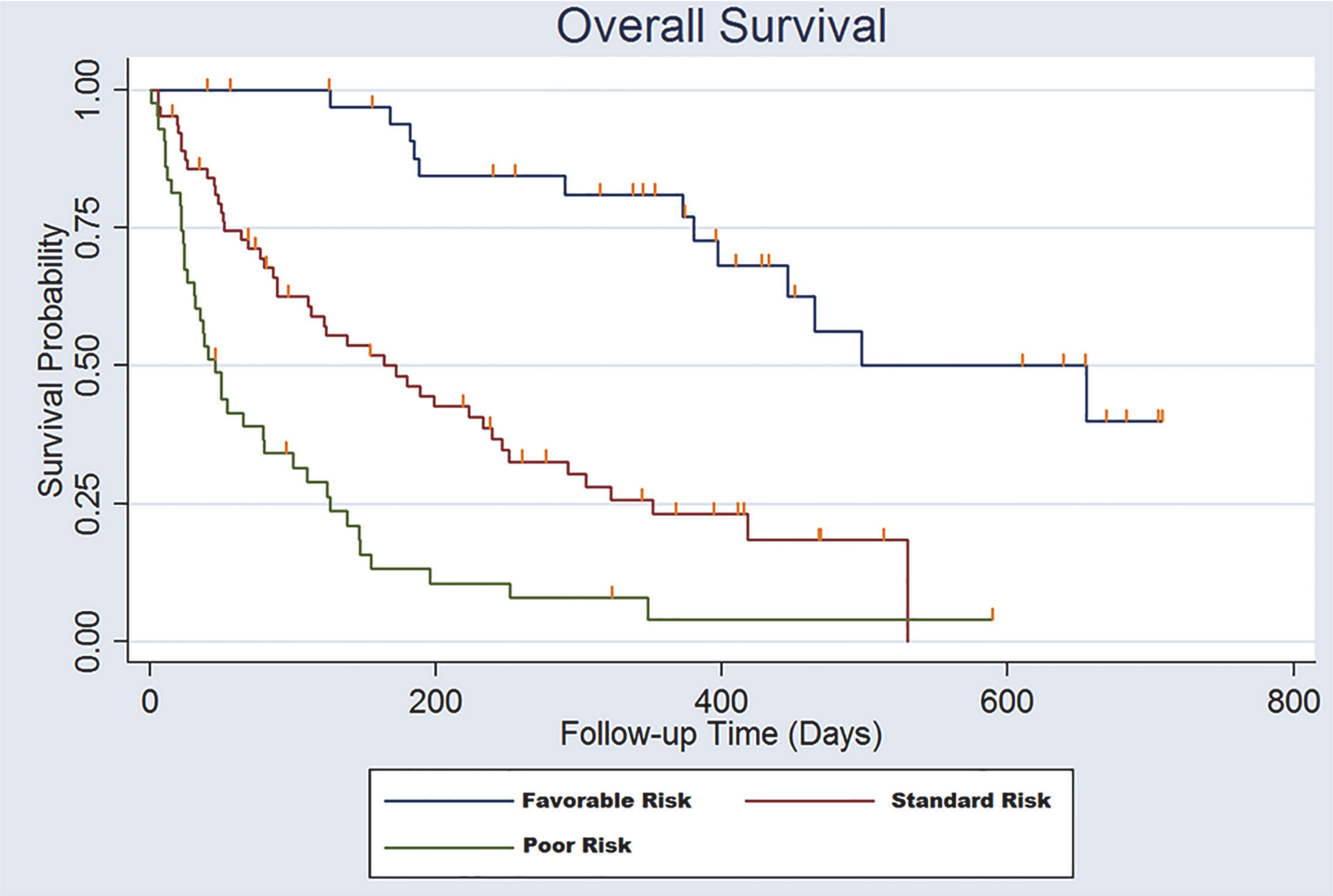


**Fig 1. Overall Survival Stratified by Risk Score.** Very low risk patients have a risk score of 0 to 1. Low risk patients have a risk score of 1.5 to 2. Intermediate risk patients have a risk score of 2.5 to 3.5. High risk patinets have a risk score of ≥4. Patients receive 1 point for serum albumin <2.4, ECOG performance status 2, ≥6 active tumors or primary site other than breast, kidney or prostate. Patients receive 2 points for ECOG performance status 3 to 4 and 0.5 points for serum albumin 2.4 to 3.3.

doi:10.1371/journal.pone.0124329.g001

The recently published TEACHH model from Harvard Medical School supplemented primary site and performance status with prior chemotherapy, prior hospitalization and liver metastases but did not include laboratory values, weight loss or clinical symptoms [29].

Likely explanations for the variance in prognostic factors identified in studies of stage IV disease include the different patient populations and significant differences in parameters collected. Some studies focused only on far advanced hospice or palliative care patients that deemphasized the contribution of tumor characteristics [30,31]. Prognostic factors identified among hospitalized stage IV cancer patients may not be generalizable to outpatients that tend to have better function and higher activity levels [14,29,30]. A unique strength of this study is that patients in this study were uniformly evaluated by a single physician rather than aggregated data from multiple providers. Therefore, our database evaluated more clinically relevant parameters than prior efforts in the field of survival prognostication in radiation oncology. Our predictive model had a robust $R^2$ coefficient of 0.50, which was significantly higher than performance status alone or the published three variable Toronto model of 0.23 [15]. Potential weaknesses of this study are the retrospective study design, heterogeneous patient population and the

relatively low ratio of events to variables analyzed. Multi-institutional prospective validation of this model relative to competing models, such as the Toronto and Harvard models is ongoing. Moreover, the performance of various predictive models should be tested in specific primary tumor types, specific metastatic sites and should include additional biomarkers, including genomics. Further use and development of predictive models could allow clinicians to better tailor supportive care and treatment for patients with stage IV cancer.

## Author Contributions

Conceived and designed the experiments: JK. Performed the experiments: JK KDG GZ. Analyzed the data: JK EC AJS SSR DY. Contributed reagents/materials/analysis tools: JK KDG GZ. Wrote the paper: JK EC SR.

## References

1. Chow E, Fung K, Panzarella T, Bezjak A, Danjoux C, et al. A predictive model for survival in metastatic cancer patients attending an outpatient palliative radiotherapy clinic. Int J Radiat Oncol Biol Phys. 2002; 53: 1291–1302. PMID: 12128132
2. Lamont EB, Christakis NA. Complexities in prognostication in advanced cancer: "to help them live their lives the way they want to". JAMA. 2003; 290: 98–104. PMID: 12837717
3. Christakis NA, Lamont EB. Extent and determinants of error in doctors' prognoses in terminally ill patients: prospective cohort study. BMJ. 2000; 320: 469–472. PMID: 10678857
4. Glare P, Virik K, Jones M, Hudson M, Eychmuller S, et al. A systematic review of physicians' survival predictions in terminally ill cancer patients. BMJ. 2003; 327: 195–198. PMID: 12881260
5. Gripp S, Mjartan S, Boelke E, Willers R. Palliative radiotherapy tailored to life expectancy in end-stage cancer patients: reality or myth? Cancer. 2010; 116: 3251–3256. doi: 10.1002/cncr.25112 PMID: 20564632
6. Guadagnolo BA, Liao KP, Elting L, Giordano S, Buchholz TA, et al. Use of radiation therapy in the last 30 days of life among a large population-based cohort of elderly patients in the United States. J Clin Oncol. 2013; 31: 80–87. doi: 10.1200/JCO.2012.45.0585 PMID: 23169520
7. Weichselbaum RR, Hellman S. Oligometastases revisited. Nat Rev Clin Oncol. 2011; 8: 378–382. doi: 10.1038/nrclinonc.2011.44 PMID: 21423255
8. Rades D, Stalpers LJ, Veninga T, Schulte R, Hoskin PJ, et al. Evaluation of five radiation schedules and prognostic factors for metastatic spinal cord compression. J Clin Oncol. 2005; 23: 3366–3375. PMID: 15908648
9. Glare P Clinical predictors of survival in advanced cancer. J Support Oncol. 2005; 3: 331–339. PMID: 16218255
10. Maltoni M, Nanni O, Derni S, Innocenti MP, Fabbri L, et al. Clinical prediction of survival is more accurate than the Karnofsky performance status in estimating life span of terminally ill cancer patients. Eur J Cancer. 1994; 30A: 764–766. PMID: 7917534
11. Maltoni M, Caraceni A, Brunelli C, Broeckaert B, Christakis N, et al. Prognostic factors in advanced cancer patients: evidence-based clinical recommendations—a study by the Steering Committee of the European Association for Palliative Care. J Clin Oncol. 2005; 23: 6240–6248. PMID: 16135490
12. Anderson F, Downing GM, Hill J, Casorso L, Lerch N. Palliative performance scale (PPS): a new tool. J Palliat Care. 1996; 12: 5–11. PMID: 8885562
13. Ma C, Bandukwala S, Burman D, Bryson J, Seccareccia D, et al. Interconversion of three measures of performance status: an empirical analysis. Eur J Cancer. 2010; 46: 3175–3183. doi: 10.1016/j.ejca.2010.06.126 PMID: 20674334
14. Barbot AC, Mussault P, Ingrand P, Tourani JM. Assessing 2-month clinical prognosis in hospitalized patients with advanced solid tumors. J Clin Oncol. 2008; 26: 2538–2543. doi: 10.1200/JCO.2007.14.9518 PMID: 18487571
15. Chow E, Abdolell M, Panzarella T, Harris K, Bezjak A, et al. Predictive model for survival in patients with advanced cancer. J Clin Oncol. 2008; 26: 5863–5869. doi: 10.1200/JCO.2008.17.1363 PMID: 19018082
16. Christakis NA, Escarce JJ. Survival of Medicare patients after enrollment in hospice programs. N Engl J Med. 1996; 335: 172–178. PMID: 8657216

17. Reuben DB, Mor V, Hiris J. Clinical symptoms and length of survival in patients with terminal cancer. Arch Intern Med. 1988; 148: 1586–1591. PMID: 3382303
18. Glare PA, Eychmueller S, McMahon P. Diagnostic accuracy of the palliative prognostic score in hospitalized patients with advanced cancer. J Clin Oncol. 2004; 22: 4823–4828. PMID: 15570085
19. Janisch L, Mick R, Schilsky RL, Vogelzang NJ, O'Brien S, et al. Prognostic factors for survival in patients treated in phase I clinical trials. Cancer. 1994; 74: 1965–1973. PMID: 8082103
20. Rosenthal MA, Gebski VJ, Kefford RF, Stuart-Harris RC. Prediction of life-expectancy in hospice patients: identification of novel prognostic factors. Palliat Med 1993; 7: 199–204. PMID: 8261188
21. Milano MT, Katz AW, Muhs AG, Philip A, Buchholz DJ, et al. A prospective pilot study of curative-intent stereotactic body radiation therapy in patients with 5 or fewer oligometastatic lesions. Cancer. 2008; 112: 650–658. PMID: 18072260
22. Kao J, Chen CT, Tong CC, Packer SH, Schwartz M, et al. Concurrent sunitinib and stereotactic body radiotherapy for patients with oligometastases: final report of a prospective clinical trial. Target Oncol 2014; 9: 145–153. doi: 10.1007/s11523-013-0280-y PMID: 23660867
23. Tree AC, Khoo VS, Eeles RA, Ahmed M, Dearnaley DP, et al. Stereotactic body radiotherapy for oligometastases. Lancet Oncol. 2013; 14: e28–37. doi: 10.1016/S1470-2045(12)70510-7 PMID: 23276369
24. Corbin KS, Hellman S, Weichselbaum RR. Extracranial oligometastases: a subset of metastases curable with stereotactic radiotherapy. J Clin Oncol. 2013; 31: 1384–1390. doi: 10.1200/JCO.2012.45.9651 PMID: 23460715
25. Kao J, Packer S, Vu HL, Schwartz ME, Sung MW, et al. Phase 1 study of concurrent sunitinib and image-guided radiotherapy followed by maintenance sunitinib for patients with oligometastases: acute toxicity and preliminary response. Cancer. 2009; 115: 3571–3580. doi: 10.1002/cncr.24412 PMID: 19536893
26. Salama JK, Hasselle MD, Chmura SJ, Malik R, Mehta N, et al. Stereotactic body radiotherapy for multisite extracranial oligometastases: final report of a dose escalation trial in patients with 1 to 5 sites of metastatic disease. Cancer. 2012; 118: 2962–2970. doi: 10.1002/cncr.26611 PMID: 22020702
27. Kao J, Darakchiev B, Conboy L, Ogurek S, Sharma N, et al. Tumor Directed, Scalp Sparing Intensity Modulated Whole Brain Radiotherapy for Brain Metastases. Technol Cancer Res Treat. 2014 Nov 26 doi: 10.7785/tcrt.2012.500426
28. Grabowski CM, Unger JA, Potish RA. Factors predictive of completion of treatment and survival after palliative radiation therapy. Radiology. 1992; 184: 329–332. PMID: 1377829
29. Krishnan MS, Epstein-Peterson Z, Chen YH, Tseng YD, Wright AA, et al. Predicting life expectancy in patients with metastatic cancer receiving palliative radiotherapy: the TEACHH model. Cancer. 2014; 120: 134–141. doi: 10.1002/cncr.28408 PMID: 24122413
30. Feliu J, Jimenez-Gordo AM, Madero R, Rodriguez-Aizcorbe JR, Espinosa E, et al. Development and validation of a prognostic nomogram for terminally ill cancer patients. J Natl Cancer Inst. 2011; 103: 1613–1620. doi: 10.1093/jnci/djr388 PMID: 21972226
31. Morita T, Tsunoda J, Inoue S, Chihara S. The Palliative Prognostic Index: a scoring system for survival prediction of terminally ill cancer patients. Support Care Cancer. 1999; 7: 128–133. PMID: 10335930
